# Vital Statistics

**Published:** 1870-08

**Authors:** 


VITAL STATISTICS.—In an article of considerable length (*N. O. Journal of Med.*), compiled with much care and labor, Dr. S. E. Chaillé presents the results of his research on the vital statistics of New Orleans. He has prepared many tables, and gathered his information from many sources; most of the paper, however, is chiefly valuable to the profession and people of New Orleans. The various tables all unite to express the result that that city has been one of the most unhealthy of the United States. It is well established that 11 deaths occur yearly in every 1000 inhabitants unavoidably; any in excess of this in healthy communities is preventable. Cities are more unhealthy than the country; but for these, 17 to the 1000 is the estimate of *necessary* mortality; yet as an average for all cities, 20 per 1000 is regarded as a fair standard of deaths, while under 20 is very healthy, and over 30 is very decidedly unhealthy.

The death-rate of New Orleans for 1856–60, five of the healthiest years, was 46.3 with, and 39.8 without the yellow fever deaths counted. The death-rate of the males, reckoned alone, was about 57, and of the females 36 per 1000, for the 46.3 of the whole population.

In the past forty years, London, Paris, and other cities, have by hygienic measures reduced the death-rate to 30 per 1000, or below that. The city of Salisbury reduced her death-rate by complete drainage of the city, 6 per 1000. The city of Ely by sanitary works shows a similar record. When Algeria was first colonized, the mortality in Mitildge was 206 per 1000, and births hardly any; but in 1868 the deaths were only 24 per 1000, and the births 39 to every 30 deaths.

He gives the annual death-rate (per 1000) of many cities and countries; those most reliable are the following: France, 23 to 24; England, 22; Massachusetts, 19.6; Vienna, 31.7; Rotterdam, 34; Paris, 25 to 29; London, 24; Boston, 23; New York, 25 to 30; Philadelphia, 20.3; Cincinnati, 24.

